# Carotid Artery Calcification: What We Know So Far

**DOI:** 10.7759/cureus.18938

**Published:** 2021-10-21

**Authors:** Madeeha Ahmed, Regina McPherson, Alexandra Abruzzo, Sneha E Thomas, Vasavi Rakesh Gorantla

**Affiliations:** 1 Family Medicine, American University of Antigua College of Medicine, Antigua, ATG; 2 Anatomical Sciences, American University of Antigua, St.John's, ATG; 3 Anatomical Sciences, St. George's University School of Medicine, St. George's, GRD; 4 Internal Medicine, University of Maryland Medical Center, Baltimore, USA; 5 Anatomical Sciences, St.George's University, St. George's, GRD

**Keywords:** risk factors, pathophysiology, pathogenesis, diagnostic method, carotid artery calcification

## Abstract

Carotid artery calcification (CAC) is a well-known marker of atherosclerosis and is linked to a high rate of morbidity and mortality. CAC is divided into two types: intimal and medial calcifications, each with its own set of risk factors. Vascular calcification is now understood to be an active, enzymatically regulated process involving dystrophic calcification and endothelial dysfunction at an early stage. This causes a pathogenic inflammatory response, resulting in calcium phosphate deposition in the form of microcalcifications, which causes plaque formation, ultimately becoming unstable with sequelae of complications. If the inflammation goes away, hydroxyapatite crystal formation takes over, resulting in macro-calcifications that help to keep the plaque stable. As CAC can be asymptomatic, it is critical to identify it early using diagnostic imaging.

The carotid artery calcification score is calculated using computed tomography angiography (CTA), which is a confirmatory test that enables the examination of plaque composition and computation of the carotid artery calcification score. Magnetic resonance angiography (MRA), which is sensitive as CTA, duplex ultrasound (DUS), positron emission tomography, and computed tomography (PET-CT) imaging with (18) F-Sodium Fluoride, and Optical Coherence Tomography (OCT) are some of the other diagnostic imaging modalities used. The current therapeutic method starts with the best medical care and is advised for all CAC patients. Carotid endarterectomy and carotid stenting are two treatment options that have mixed results in terms of effectiveness and safety. When patient age and anatomy, operator expertise, and surgical risk are all considered, the agreement is that both techniques are equally beneficial.

## Introduction and background

Carotid Artery Calcification (CAC) has been recognized as a symptom of aging for decades. Vascular calcification, once thought to be a passive and degenerative process, is now widely recognized as an active and self-regulating process involving complex cellular and enzymatic components. Vascular calcification is well documented to play a role in the progression of atherosclerosis, and it can be found in 80-90% of atheromas [1]. Calcification can develop in the medial and intimal layers of smaller elastic vessels, such as the intracranial and cervical carotid arteries [2]. Atheromatous plaques arise because of intimal calcification, which can lead to vascular stenosis, occlusion, or other secondary degenerative alterations [2]. Symptomatic carotid artery stenosis, ischemic stroke, blindness, cognitive impairment, and severe morbidity and mortality are all possible outcomes of such changes. Current research on CAC epidemiology and risk factors, pathogenesis, and the best clinical diagnostic and management approaches are reviewed in this article.

## Review

In a cohort analysis of 1132 patients, predominant intimal calcification was found in 30.9% and medial calcification in 46.9%, with few isolated risk factors in the middle [3]. Overweight and obesity are major determinants of metabolic syndrome, an all-too-common and all-too-serious clinical and public health challenge. Clinicians have traditionally evaluated each of the major risk factors contributing to metabolic syndrome on an individual basis. There is evidence, however, that the risk factors are more than additive. Age, male gender, positive family history, and higher pulse pressures all contribute to the occurrence of CAC linked with both types of calcifications [1]. Carotid artery calcification was found in 75% of males over 75-years-old and 62% of females over 75-years-old in a comprehensive review [4]. Clinical research in recent years has found that people of Asian and Caucasian descent had a higher prevalence of cerebral arterial calcification [5-7]. As Wu et al. point out, the Rotterdam research, the biggest population-based cohort study in a general community with 2,495 participants, backs this up [5,8]. The Rotterdam study established carotid artery calcification as a substantial risk factor for stroke in the White population after assessing cerebral carotid artery calcification volume on CT scans in 2,323 stroke-free older Caucasian patients. The findings also revealed that cerebral carotid artery calcification contributes more to all-cause strokes than large-artery atherosclerosis, contrary to popular belief [5,9].

Diabetes, hypercholesterolemia, smoking, and hypertension are all known risk factors for the development of cardiovascular and systemic atherosclerotic disease in CAC [5]. Smoking and high blood pressure are two risk factors for dominant intimal calcification. Hypertension damages endothelial cells, resulting in pro-inflammatory activity and decreased vascular contractility, which leads to atherosclerosis.

Type 2 diabetes mellitus (T2DM), chronic kidney disease (CKD), end-stage renal disease (ESRD), and osteoporosis, all of which have an inherent calcium-phosphate imbalance, are major risk factors for Monckeberg medial calcification. Men and T2DM patients are more likely to develop this form of vascular calcification. Patients with ESRD who showed medial calcification were younger, had a higher prevalence of the calcium-phosphate problem and were hemodialysis dependent [10]. Calciphylaxis is a specific manifestation of medial calcification reported in hemodialysis patients [5,10-15].

Pathogenesis and pathophysiology

Some similarities and variations exist between the mechanisms underlying intimal and medial calcification. Microcalcifications, also known as spotty calcifications, occur during the first stage of intimal calcification, which is characterized by an acute inflammatory response. Several investigations have established that these little calcium deposits (0.5 to 15 m) are an insidious source of plaque instability, since they have been found in high-risk, unstable, and ruptured plaques in abundance [2,16-18].

Endothelial dysfunction of the vascular intima results in the deposition of low-density lipoprotein particles (LDLs) within the intimal layer of the artery, which starts the pathological process of microcalcification development. The presence of low-density lipoprotein cholesterol (LDLC), oxidative stress, and other harmful elements cause an inflammatory reaction within the artery wall, resulting in the release of cytokines including tumor necrosis factor (TNF) and Interleukin-1ß (IL-1ß) [2,19]. Monocytes, vascular smooth muscle cells (VSMCs), and other inflammatory cells in the intima migrate and proliferate in response to these cytokines [2,19]. When monocytes attempt to phagocytize deposited LDLs, they form lipid-laden macrophages, also known as foam cells. The oxidation of lipoproteins is an unpleasant and unanticipated side effect of this. These oxidized lipids are particularly hazardous to macrophages, causing apoptosis or necrosis [16]. When macrophages die, phagocytized material is released from the cells in vesicles, which act as a nidus for calcium phosphate deposition. Macrophages and VSMCs release vesicles during intimal calcification, while VSMCs release them during medial calcification [10]. A plaque forms when inflammatory material microcalcifies and develops a necrotic core beneath it.

The process of artery damage, necrosis, and surface microcalcification continue indefinitely if acute inflammation persists, and the vascular layer never entirely recovers. This creates an intrinsically unstable plaque that is prone to rupture and thrombus development [16-18].

If the inflammation eventually subsides, plaque reformation and stability can take place. Chondrocyte-like VSMCs direct a regulated mineralization process and the production of stabilizing macrocalcification, which drives plaque remodeling [16].

This calcification process resembles chondrogenesis and osteogenesis in appearance [2,5,19]. Matrix Gla Protein (MGP), Osteocalcin (OC, also known as bone Gla protein), Bone Sialoprotein (BSP), Bone Morphogenetic Proteins (BMP) 2, 4, 6, and 7, Osteopontin (OPN), and Osteonectin (ON) are only a few of the proteins that have been linked to the osteogenic transformation of VSMCs [5,19]. BMP2, BMP4, and BMP6 are the proteins that are thought to be most important in atherogenic calcification [12,17,19]. BMPs are members of the transforming growth factor-beta (TGFß) family and are required for appropriate bone formation, angiogenesis, vascular homeostasis, and a variety of physiological activities [12,17,19].

BMP2 and BMP4 cause medial VSMCs and macrophages to develop into osteoblast-like phenotypes during medial calcification. The vascular extracellular matrix is then transformed into a cartilaginous matrix by these cells. The matrix of the cartilage is calcified, and hydroxyapatite crystals develop. Endochondral ossification of bone is the same process [2,5,12]. These crystal aggregates eventually merge to generate macro-calcification calcified sheets or plaques.

Because BMPs are tightly regulated and can be suppressed by a variety of modulator proteins, including Osteopontin, Gremlin, Noggin, and uncarboxylated Matrix Gla Protein (MGP), the progressive mineralization that generates macro-calcification that stabilizes the plaque and prevents additional inflammation [5,12,16].

Clinical Manifestations

There are no specific clinical indications for carotid artery calcification. On the other side, this phenomenon is sometimes misconstrued as a proxy for atherosclerosis, leading to clinical indications being misinterpreted as atherosclerosis issues. Because it can be a marker of luminal stenosis, arterial calcification is associated with ischemic symptoms. Signs and symptoms arise when an artery is highly stenotic or completely occluded. The most common signs and symptoms include bumps, amaurosis fugax, contralateral weakness or numbness of an extremity or face, with significant sparing of the forehead, dysarthria, aphasia, transient ischemic attack (TIA), or ischemic stroke.

Symptomatic vs Asymptomatic Carotid Artery Plaques

Shaalan et al. used Spiral CT to measure the percent plaque calcification area and performed an assessment of carotid plaque calcification. They discovered that asymptomatic patients had two times more plaque calcification area than symptomatic patients (48% +/-19% versus 24% +/-20%; P0.05) [20]. Shaalan et al. concluded that higher carotid artery plaque calcification is associated with plaque stability and could serve as a quantitative measure for cerebrovascular ischemia event risk based on this and other findings throughout their investigation [20]. Hunt et al. found that patients with calcified carotid plaques had fewer symptoms of stroke and transient ischemic attack (P = 0.042) than patients with non-calcified carotid plaques [21].

Shi et al. recently discovered that asymptomatic patients exhibited plaque calcification areas that were two times larger than symptomatic patients [2,22]. Intraplaque bleeding and ulceration were frequently found in plaques with a greater calcification volume and numerous calcifications. Multiple calcifications may trigger a stronger inflammatory response, increasing the risk of the plaque becoming ulcerated and hemorrhagic, according to one theory. While the authors acknowledged that plaque calcification and plaque stability are linked, they were skeptical about the information's utility in forecasting future cerebrovascular accidents [2,23-26]. Asymptomatic carotid plaques are more calcified than symptomatic plaques, according to Wu et al., and extracranial carotid plaque calcification is linked to plaque stability [5]. They also disclosed a study that used carotid-femoral pulse wave velocity and 24-hour ambulatory pulse pressure to quantify arterial stiffness. There was increased stiffness in major cerebral arteries in patients without stroke and with asymptomatic intracranial atherosclerosis, according to this study [27].

According to the findings from the various sources, calcified carotid artery plaques may be more stable than non-calcified carotid artery plaques. The findings of Fisher et al. and Wu et al., on the other hand, are in opposition to this [5,28]. Patients who were asymptomatic were compared to patients who had stroke symptoms ipsilateral or contralateral to the plaque by Fisher et al.. They discovered that asymptomatic, ipsilateral symptomatic, and contralateral symptomatic patients all had similar plaque calcification averages (16%, and 15%, respectively; P0.001), leading them to conclude that the extent of carotid plaque calcification had no relation to symptoms [28].

Stroke and Transient Ischemic Attack

There was a decreased incidence of stroke and TIA in patients with carotid plaques composed of large calcific granules, compared to those without calcification (P = 0.021) and large calcifications were inversely related to plaque rupture [2,21]. Hunt et al. also found that 52 out of the 142 patients who had a TIA or stroke, 65% (n = 34) did not have any carotid plaque calcification, while 35% (n = 18) had a calcified plaque (P < 0.042) [21]. In contrast, Golüke et al. found that intimal and medial intracranial carotid artery calcifications were significantly associated with stroke [adjusted odds ratio (OR) 1.84, 1.88, and 2.88, respectively], and the severity of calcification in both intimal and medial layers also had significant associations with stroke and myocardial infarction [29]. Furthermore, a Korean study involving 445 patients determined that higher carotid siphon calcification scores were associated with higher rates of lacunar infarction [5,30].

Several studies correlated carotid artery calcifications and cardiovascular disease. Dominant intimal calcifications were significantly associated with myocardial infarction (adjusted OR 2.27 and 4.45, respectively) [29]. Patients with carotid plaques composed of large sheets of calcium were more likely to have coronary artery disease (P < 0.0333) [21]. Shaalan et al. concluded that there was no difference between the prevalence of atherosclerotic risk factors between symptomatic and asymptomatic groups, except for hypercholesterolemia, which had a higher prevalence in the group with symptomatic carotid artery plaques (36% vs 26%; P < 0.05) [20].

Other well-researched clinical manifestations associated with intracranial carotid artery calcification include deep cerebral microbleeds and white matter hyperintensities, as described by Wu et al.. cognitive decline and dementia are well studied as proposed clinical manifestations of carotid artery calcification. Suggested mechanisms relate to decreased cerebral blood flow regulation leading to cognitive deficits. Chu et al. described a significant negative correlation between carotid artery calcification scores and cognition (R = -0.359, P < 0.001), in a study of 102 patients with confirmed carotid artery calcification using color doppler ultrasound, multi-detector row spiral CT angiography, and MRI scanning [31]. Furthermore, a cross-sectional study of 2,414 non-demented people in the Rotterdam Study, followed by a subsequent longitudinal study, both demonstrated a cognitive decline in individuals with larger intracranial carotid artery calcification volume [5,8,32].

Diagnostic methods

Diagnostic techniques for carotid artery calcification have advanced in sophistication over time. Improved diagnostic tools aid in the early detection of potentially life-threatening disorders as well as clinical decision-making. Although duplex ultrasonography (DUS) is often the first diagnostic test indicated for carotid artery stenosis evaluation, confirmatory imaging modalities such as magnetic resonance angiography (MRA) or computed tomography angiography (CTA) is currently used. These diagnostic tests are used to guide therapeutic measures, such as procedure planning. Another regularly scheduled screening technique that has been shown to detect calcified atheromas in the carotid artery is panoramic radiography. The frequency of confirmed accidental carotid artery calcification identified on panoramic radiography ranges from 2% to 5%. [33]. Duplex ultrasound is frequently performed after this test, and the results are validated with computed tomography angiography or magnetic resonance angiography. More advanced imaging technologies, including optical coherence tomography (OCT), photoacoustic tomography, and infrared thermography, have been developed to characterize plaques [34]. PET-CT imaging using (18)F-Sodium Fluoride was originally developed to detect calcifying metastases, but it is currently utilized to detect and quantify microcalcification in atherosclerotic plaques. Its relationship with cardiovascular risk factors makes it a helpful noninvasive approach for determining the amount and severity of carotid artery calcification [35-37].

Duplex Ultrasound (DUS)

Duplex ultrasonography (DUS) is one of the most used and well recommended initial diagnostic tests for carotid artery disease since it is non-invasive, low-cost, and accurate. It enables for direct visualization of vessel shape as well as flow measurement. It has a sensitivity of 86.4 for 50% stenosis and 92.1 for 100% stenosis, as well as a specificity of 90.1 for 50% stenosis and 89.5 for 100% stenosis [33]. DUS cannot tell the difference between high-grade stenosis and total occlusion, which is a disadvantage [38].

Computed Tomography Angiography (CTA) and Magnetic Resonance Angiography (MRA)

The use of intravenously supplied iodine contrast medium and complete vascular visualization to diagnose carotid artery disease using Computed Tomography Angiography (CTA) allows for the assessment of stenotic degree. This diagnostic modality allows for plaque composition characterization and calcification score computation in the carotid artery. CTA has a sensitivity of 89 for 50% stenosis and 99% for 99% stenosis, according to Anderson et al. [39]. The specificity reported was 91% for 50% stenosis and 99% for 100% stenosis [39]. CTA was proven to be nearly 100% accurate for total blockage and stenosis between 0 and 29% [39]. CTA and magnetic resonance angiography (MRA) are both sensitive, but CTA has a greater specificity [34]. The requirement for intravenous contrast injection and radiation exposure are both disadvantages of CTA [38]. MRA is a non-invasive way of detecting stenotic vessels that can be utilized with or without contrast. Similarly, to DUS, MRA may exaggerate the degree of stenosis; consequently, combining DUS and MRA is more accurate than doing either test alone [38].

PET-CT imaging with (18)F-Sodium Fluoride

PET-CT imaging with (18)F-Sodium Fluoride (18F-NaF) is a molecular imaging technique that uses an 18 Fluoride-labeled sodium fluoride radiotracer to detect areas of necrotic inflammation and metabolic activity in atherosclerotic plaques. Derlin et al. found a significantly significant connection (r = 0.85; P0.0001) between the occurrence and amount of artery wall calcification and 18F-sodium fluoride absorption [36]. When compared to other PET radiotracers, 18F-sodium is cleared from circulation faster, has no superfluous soft-tissue uptake, and has significantly higher absorption in important artery walls than 18F-FDG [16, 35-37]. Because CT scans can only identify large areas of calcification, they are more likely to overlook newer and smaller calcifications. With the help of x-ray attenuation, 18F-NaF was able to detect microcalcifications not evident on CT [35-37, 40]. There was also a statistically significant relationship between tracer uptake in the common carotid arteries and cardiovascular risk variables such as age, male sex, hypertension, hypercholesterolemia, and cumulative smoking exposure (P0.0001) [35-37,41-45].

Optical Coherence Tomography (OCT)

The highest resolution of any intravascular imaging method is optical coherence tomography (OCT), which can be used to classify the volume of calcification. This enables the visualization of plaque shape, calcification features, and potential vulnerability in more detail. It is extremely sensitive and specific for assessing carotid artery calcification [34]. An intravascular OCT catheter was used to see the artery wall, and the imaging results were consistent with those obtained from histological inspection. When OCT was first employed during carotid artery stenting, it successfully detected fibrocalcific carotid plaque disruption, thrombosis, and plaque protrusion in 17 individuals [34]. After angioplasty and stenting, OCT has been successfully used to detect possible stenting problems such as plaque ulceration and thrombus development. However, because of its invasiveness, further research is needed before it may be used more widely [34,46].

Current management strategies

Medical Management

The goal of medical care in individuals with carotid atherosclerotic disease is to reduce the number of cerebrovascular episodes. Chimowitz et al. observed that patients who underwent a stenting surgery with intensive medical therapy had a 20.0% one-year rate of stroke or death, compared to 12.2% in patients who received aggressive medical management alone (P = 0.009) [47]. They determined that medical care alone was superior to medical management combined with percutaneous angioplasty and stenting in terms of lowering the risk of stroke [47].

Risk factor adjustment, antithrombotic treatment, and statin medication are all recommended for optimal management. Regardless of symptoms or stenosis severity, this is suggested for all individuals with carotid stenosis [48-50].

Modification of risk factors can include the following: 1) A systolic blood pressure target of 140/90 mmHg, 2) LDL cholesterol reduction, 3) smoking cessation and 4) moderate to vigorous-intensity aerobic physical exercise of at least 40 minutes per day, three to four days per week [38, 48, 50]. Clinicians should prescribe a low, moderate, or high-intensity statin depending on the patient's 10-year risk of atherosclerotic cardiovascular disease [38, 48]. Aspirin 325 mg per day and an antiplatelet medication are used as antithrombotic treatment [38, 47].

Carotid Endarterectomy and Carotid Stenting

Carotid endarterectomy is a treatment that is performed to reduce the risk of TIA and stroke in those who have carotid atherosclerosis. A qualified surgeon should execute the surgery, which involves accessing the common and/or internal carotid arteries via the sternocleidomastoid muscle and removing atheromatous plaque material [51]. Plaque clearance should improve cerebrovascular perfusion by increasing the luminal size. Even though the technique has been used for decades, the inherent dangers of the surgery need clinicians and patients to carefully weigh the benefits and risks before opting to undergo it. The North American Symptomatic Carotid Endarterectomy Trials (NASCET) and the Asymptomatic Carotid Artery Stenosis (ACAS) trials for endarterectomy were conducted in 1987, and the current treatment recommendations and indications for endarterectomy are based on these trials [49, 51-52]. Carotid endarterectomy (CEA) is advised for symptomatic patients (those who have had a TIA or stroke in the past) if carotid stenosis is 50% or greater, and it must be conducted soon after the onset of symptoms to be useful [51]. CEA is suggested for carotid stenosis of 70% or greater in asymptomatic patients [49,51].

The current standard endovascular therapy intervention for carotid atherosclerotic disease is carotid stenting [48, 53]. For patients who are unable to undergo endarterectomy, stenting is an option [48]. There are several carotid vascular access and stent implantation techniques available, each with its own set of risks and benefits [48]. Stenting can be done percutaneously with a transfemoral approach or by a tiny incision in the neck with a transcarotid technique [48]. Patients aged 80 and up have a higher chance of negative outcomes after transfemoral stenting, however, this is not the case with the transcarotid method [48,54]. Several strategies are employed to prevent embolic stroke during the stenting procedure, however, there is no conclusive evidence that one method is better than the others [48].

Muller et al. conducted a systematic review that compared the safety and efficacy of stenting versus carotid endarterectomy in 5,396 participants with symptomatic and asymptomatic carotid artery stenosis. The primary outcome was death or stroke occurring up to 30 days after treatment. Up to 30 days after stenting, there was a significantly increased risk of death or stroke [OR 1.70, 95% confidence interval (CI) 1.31 to 2.19; P 0.0001). [54]. Data from six of the trials revealed that participants over the age of 70 were twice as likely to die or have a stroke up to 30 days after stenting (OR=2.23, 95 % CI 1.61 to 3.08; P0.0007) [54]. However, after four years of follow-up data, it was determined that stenting and endarterectomy were equally effective in the long term in preventing recurrent stroke [54]. CAC risk factors vary depending on whether the calcification is intimal or medial dominant. There were 37 studies used in this review (Table 1).

**Table 1:** Risk factors, pathogenesis, clinical manifestations, diagnostic techniques, and management of carotid artery calcification. Portrait of the Risk factors, pathogenesis, clinical manifestations, diagnostic techniques, and management of carotid artery calcification. AIS: Acute ischemic stroke, AMI: Acute myocardial infarction, BCP: Basic calcium phosphate,  BMP-2: Bone morphogenetic protein 2, BMP-4: Bone morphogenetic protein 4, CEA: Carotid endarterectomy, cf-PWV: carotid-femoral pulse wave velocity, cMGP: Carboxylated Matrix Gla Protein, CT: Computed tomography, CTA: Computed tomographic angiography, ECM: Extracellular matrix, ERK1/2: Extracellular signal-regulated kinase ½, (18)F-FDG: (18)F-fluorodeoxyglucose, (18)F-NaF: (18)F-sodium fluoride, GFR: Glomerular filtration rate, HR: Hazard ratio, IAC: Intracranial arterial calcification, ICAC: Internal carotid artery calcification, IL-1B: Interleukin 1 beta, IL-8: Interleukin 8, JNK: c-Jun N-terminal kinase, MI: Myocardial infarction, OR: Odds ratio, PET: Positron emission tomography, PET/CT: Hybrid positron emission tomography and computed tomography, PKC: Protein Kinase C, PTAS: Percutaneous transluminal angioplasty and stenting, RR: Relative risk, SAP: Stable angina pectoris,TIA: Transient ischemic attack, TF-CAS: Transfemoral carotid artery stenting, TNFa: Tumor necrosis factor alpha, UAP: Unstable angina pectoris

Risk factors, pathogenesis, clinical manifestations, diagnostic techniques and management of carotid artery calcification					
	Author	Country	Study population	Findings	Conclusion
1	Vos et al., 2018 [3]	Netherlands	1132 patients with AIS	Dominant intimal and medial calcification was present in 30.9% and 46.9% of patients, respectively. Pulse pressure, age, and family history were risk factors for both. Adjusted risk factors for intimal calcification were smoking (OR: 2.09) and hypertension (OR:2.20), while medial calcification risk factors were diabetes mellitus (OR: 2.39) and history of vascular disease (OR:2.20)	Different risk factors exist for intracranial ICAC depending on localization to the medial or intimal layer
2	Song et al., 2020 [4]	Meta-analysis	515 studies regarding atherosclerotic disorders	For people between the ages of 30-79 years, prevalence of increased carotid intima-media thickness was 27.6%, carotid plaque was 21.1%, and carotid stenosis was 1.5%, all of which increased with age. Common risk factors for increased carotid thickness and carotid plaque were smoking, diabetes, and hypertension	Carotid atherosclerosis is highly prevalent around the world
3	Chen et al., 2006 [6]	China	490 patients referred for brain CT	60% of intracranial calcifications were ICAC. Age (RR= 2.795), history of ischemic stroke (RR= 3.915) and white blood cell count (RR= 1.107) were all independently associated with IAC. Many other risk factors such as hypertension, heart disease and ischemic stroke (p<0.001) were significantly prevalent in patients with artery calcification	IAC is associated with white blood cell count, age, and history of ischemic stroke
4	Mak et al., 2009 [7]	China	60 patients with TIA or ischemic stroke	Intracranial internal carotid artery was the most severely affected vessel in patients. Diabetes mellitus and age were both significant risk factors for ICAC among patients, p=0.004 and p=0.02 respectively	Internal carotid artery was the most severely calcified vessel, and IAC was associated with diabetes mellitus and age
5	Bos et al., 2012 [8]	Netherlands	2495 patients who underwent brain CT	ICAC was present in 82.2% of patients. Excessive alcohol consumption (OR: 1.74) and smoking (OR: 1.72) were strong risk factors of ICAC for men; diabetes (OR: 2.02) and hypertension (OR: 1.79) were risk factors for women. Age was a risk factor in both men and women	ICAC is very common and risk factors for ICAC differ in men and women
6	Bos et al., 2014 [9]	Netherlands	2323 patients who underwent brain CT	Of patients who suffered from stroke (n=91), 74 were ischemic strokes. 75% of all strokes were related to ICAC. Increased ICAC volume was correlated with increased risk of stroke (HR: 1.43)	Intracranial atherosclerosis increases risk of stroke in white population
7	Nigwekar et al., 2017 [11]	USA	40 patients receiving hemodialysis	Patients with calciphylaxis had a lower fraction of carboxylated MGP (cMGP) compared to controls (p=0.003). Vitamin K deficiency was associated with lower cMGP (p=0.04), and low cMGP was correlated with an increase in calciphylaxis risk	Vitamin K deficiency reduced cMGP concentration and may have a role in calciphylaxis
8	Bugnicourt et al., 2009 [14]	France	340 patients with AIS	IAC in stroke patients (n=259) was associated with carotid atherosclerosis >50%, age and GFR. Calcification in non-stroke patients (n=103) was associated with age, arterial hypertension and GFR	Risk factors for IAC were present in patients with and without ischemic stroke, while the frequency of calcification was greater in stroke patients
9	Power et al., 2011 [15]	United Kingdom	2225 patients receiving hemodialysis	IAC was seen in 95% of patients with ischemic stroke and 83% of patients with no stroke (p=0.02). Age (p<0.001), hemodialysis vintage (p<0.001) and serum phosphate (p<0.05) increased severity of IAC. High grade IAC was significantly correlated to worse survival (p=0.008)	IAC is especially prevalent in hemodialysis patients with AIS, and its severity is significantly associated with vascular calcification risk factors
10	Sakaguchi et al., 2016 [18]	Japan	98 patients with acute coronary syndrome	Patients with plaque rupture (n=38) had higher frequency and significantly more spotty calcifications (p=0.006 and p=<0.001) than non-plaque rupture cohort (n=60). Thin-capped fibro-atheroma (p=0.012) and macrophage infiltration (p=0.022) were significantly higher in plaque rupture group, along with largest arc and minimum depth of calcification from luminal surface. Presence of spotty calcification (p=0.030) and age (p=0.008) were independent risk factors for plaque rupture	There are characteristics and positional relationships of spotty calcification plaque ruptures in patients with acute coronary syndrome
11	Dhore et al., 2001 [19]	Netherlands	42 patients with atherosclerotic plaques	Calcified atherosclerotic plaques resulted in upregulation of BMP-2, BMP-4, osteopontin and constitutive reactivity of matrix Gla protein, osteocalcin, and bone sialoprotein. Non-diseased aortas and early atherosclerotic lesions demonstrated sustained reactivity and absence of BMP-2, BMP-4, osteopontin and osteonectin. In advanced lesions, osteoprotegerin and its ligand were present bone structures and ECM around calcium deposits, respectively	There is a tight regulation of the expression of bone matrix proteins during atherosclerotic calcification
12	Shaalan et al., 2004 [20]	USA	48 carotid bifurcation plaques	Degree of stenosis in symptomatic plaques and asymptomatic plaques was 76% and 82%, respectively (p=0.05). Plaque area calcification was significantly greater (p<0.05) while macrophage infiltration was significantly less (p<0.03) in asymptomatic plaques compared with symptomatic. Plaque calcification and macrophage infiltration were inversely related in critical carotid stenosis (p<0.001)	Symptomatic plaques are less calcified and also more inflamed, as determined by macrophage infiltration, compared to asymptomatic plaques
13	Hunt et al., 2002 [21]	USA	142 carotid endarterectomy plaques	Patients with calcification of carotid plaques developed fewer symptoms of stroke and TIA than those without (p=0.042). Stroke and TIA prevalence was reduced in patients with large plaque granules (p=0.021). Presence of bone was directly correlated with sheet like calcifications (p=0.0001), diabetes (p<0.01) and coronary artery disease (p<0.01). Bone formation was inversely related to ulcerated lesions (p=0.048)	Patients with greater calcification of carotid plaques are likely to have bone formation, devoid of ulceration and hemorrhage, but remain asymptomatic
14	Miralles et al., 2006 [22]	Spain	26 patients with internal carotid artery stenosis	Asymptomatic patients had significantly higher calcium content compared to symptomatic patients (p=0.04). Plaque calcification was observed more frequently in asymptomatic patients (p=0.006), while posterior calcification or no calcification was observed more in symptomatic cohort (p=0.021)	Lower calcium content and posterior/basal distribution of calcium on carotid plaques are associated with greater incidence of neurological symptoms
15	Nadra et al., 2005 [23]	United Kingdom	10 In-vitro human-monocyte derived macrophages	Presence of basic calcium phosphate (BCP) microcrystals induced macrophage secretion of proinflammatory cytokines, TNFa, IL-1B, IL-8. Cytokines activated endothelial cells and promoted capture of leukocytes. TNFa resulted in stimulation of PKC and ERK1/2 and JNK depending on upstream activation of PKC	Calcification is not a passive effect of chronic inflammatory disease
16	Mizukoshi et al., 2013 [24]	Japan	187 patients with acute MI, unstable or stable angina pectoris	Arc, area, and length of calcium deposit were significantly smaller in patients with AMI and UAP (p<0.001), while the number of large calcium deposits was lower (p<0.001) compared to SAP. Plaque rupture was correlated with number of spotty calcium deposits (r=0.479, p<0.001) and inversely related to large calcium deposits (r=-0.219, p=0.003). Calcium was more superficial in AMI and UAP groups (p<0.001)	Calcium deposits were significantly spottier and more superficial in patients with AMI and UAP
17	Lin et al., 2017 [25]	China	142 carotid artery plaques	28.2% of plaques had intra-plaque hemorrhage and greater prevalence of calcification compared to those without (p=0.005). Multiple calcifications (OR: 10.1), surface calcification (OR: 29.4) and mixed calcification (OR: 27.9) were strongly associated with intra-plaque hemorrhage (all p<0.05)	Quantity and location of calcification may play a role in the occurrence of intra-plaque hemorrhage
18	van den Bouwhuijsen et al., 2015 [26]	Netherlands	329 patients, 611 carotid arteries with plaques	Increased calcification volume was associated with decreased presence of lipid core (OR: 2.04), especially in those with high degree of stenosis. No significant difference between calcification volume and hemorrhage on degree of stenosis	Plaques with increased calcification contain more hemorrhagic components, but not lipid core
19	Zhang et al., 2011 [27]	China	270 patients with untreated hypertension	Significant difference in carotid-femoral pulse wave velocity (cf-PWV) between patients with normal vessels (n=162), with stenosis or calcification (n=82) and with lesions (n=26). cf-PWV (OR: 1.51) and 24h pulse pressure (OR: 1.46) were independently correlated with intracranial large artery disease	cf-PWV, 24h pulse pressure, and arterial stiffness are useful in identifying likelihood of intracranial large artery disease
20	Fisher et al., 2005 [28]	USA	241 patients with carotid artery plaques	Ulceration was more prevalent in plaques from symptomatic patients than asymptomatic (p<0.001). There was no significant difference in ulceration frequency between plaques associated with contralateral and ipsilateral symptoms. Patients with ipsilateral symptoms and ulceration most presented with thrombus in plaques	Symptomatic patients had increased frequency of carotid plaque thrombosis and ulceration. Thrombus was associated primarily with ipsilateral symptoms and plaque ulceration
21	Golüke et al., 2020 [29]	Netherlands	1992 patients with ICAC or basilar artery calcifications	Patients predominantly had intracranial ICAC (± 95%). Age (p<0.001), diabetes mellitus (medial ICAC p=0.004), hypertension (intimal ICAC p<0.001, basilar artery p=0.019), and smoking (intimal ICAC p=0.008) were significant risk factors for IAC. Intracranial ICAC were associated with stroke while intimal calcifications with both stroke and MI	IAC was highly prevalent and risk factors are differentially related to medial or intimal ICAC
22	Hong et al., 2010 [30]	South Korea	445 patients without large intracranial lesions	Group I (n=328), no lacunar infarctions, Group II (n=94), 1-3 infarctions, and Group III (n=23), 4+ infarctions, all had significantly different total carotid siphon calcification scores (p<0.05). Greater prevalence of lacunar infarction was associated with higher calcification scores, and age, hypertension and calcification were high risk factors for lacunar infarction (p<0.05)	Carotid siphon calcification was associated with lacunar infarction
23	Chu et al., 2019 [31]	China	102 patients with carotid artery stenosis	Calcification score and cognitive scores were negatively correlated in patients other than postoperative patients (p<0.001). Calcification score was significantly associated with wall area (p=0.042), total area of blood vessels (p=0.017) and plaque burden (p=0.003). Calcification score was also predictive of carotid plaque lipid-rich necrotic nucleus (p=0.029) in preoperative patients	Greater calcification scores were associated with worsened cognitive scores and can be used for early screening of cognitive impairment
24	Bos et al., 2015 [32]	Netherlands	2364 patients who underwent brain CT	Higher risk of dementia was associated with larger calcification volume in aortic arch, extracranial vessels, and intracranial carotid arteries. Only extracranial carotid artery calcification was significantly associated with dementia (HR: 1.37). Larger calcification volumes were associated with cognitive decline	Atherosclerosis is associated with higher risk of dementia and cognitive decline, particularly in extracranial carotid arteries
25	Huston et al., 2000 [33]	USA	382 patients with internal carotid artery stenosis	Internal carotid artery stenosis of 70% or more displayed a peak systolic velocity of at least 230cm/s, sensitivity of 86.4%, specificity of 90.1%, positive predictive value of 82.7%, negative predictive value of 92.3% and accuracy of 88.8%. Additionally, it had an end diastolic velocity of at least 70cm/s and internal carotid artery:common carotid artery ratio of at least 3.2	Carotid artery stenosis of 70% or more can be accurately diagnosed using ultrasonographic criteria
26	Derlin et al., 2010 [35]	Germany	75 patients who underwent (18)F-NaF PET/CT	Arterial calcification was observed in 63 patients. While radiotracer was localized to calcifications, only 12% of all arterial calcification sites had notable accumulation of radiotracer uptake	(18)F-NaF PET/CT can be used to visualize deposition in arterial walls
27	Derlin et al., 2011 [36]	Germany	269 patients who underwent (18)F-NaF PET/CT	Radiotracer uptake was significantly associated with patient age (p<0.0001), male sex (p<0.0001), hypertension (p<0.002), and hypercholesterolemia (p<0.05). Calcified plaques were significantly associated with these same risk factors and diabetes (p<0.0001), smoking (p=0.03) and history of cardiovascular event (p<0.01). Radiotracer uptake and number of cardiovascular risk factors were significantly related (p<0.0001)	Carotid artery radiotracer uptake is a way to measure calcifying carotid plaques and is associated with cardiovascular risk factors
28	Derlin et al., 2011 [37]	Germany	45 patients (18)F-FDG PET vs. (18)F-NaF PET/CT)	(18)F-NaF uptake (n=27) was seen at 105 sites, and there was co-localization of radiotracer to calcified atherosclerotic lesions in 77.1% of the sites. (18)F-FDG uptake (n=34) was observed in 124 sites, and there was co-localization of radiotracer in only 14.5% of calcified lesions	(18)F-NaF and (18)F-FDG PET/CT may be used for evaluation of pathology in atherosclerotic lesions
29	Anderson et al., 2000 [39]	Canada	40 patients with TIA or stroke	Computed tomographic angiography (CTA) had nearly 100% accuracy when detecting mild carotid artery stenosis. For stenosis >50%, CTA had a sensitivity of 89%, specificity of 91% and accuracy of 90%. For stenoses 50-69% or 70-99%, CTA was much less sensitive	CTA can be used for detection and examination of carotid occlusions if stenosis is in the 0-29% or >50% range
30	Hop et al., 2019 [40]	Netherlands	23 carotid artery plaques	Average (18)F-NaF uptake was similar in culprit and non-culprit carotid plaques. A median of 10% of CT calcification of the volume of interest had increased (18)F-NaF uptake. A median of 35% of (18)F-NaF PET volume of interest displayed calcification on CT	There was similar uptake in (18)F-NaF in culprit and non-culprit plaques and (18)F-NaF PET is useful in a different stage of the calcification process
31	Dweck et al., 2011 [41]	United Kingdom	199 patients with and without aortic valve disease	(18)F-NaF uptake was higher in patients with coronary atherosclerosis (n=106) compared to control (p=0.003). (18)F-NaF was positively correlated with calcium score (p<0.001), and patients with increased (18)F-NaF had higher incidence of prior cardiovascular events (p=0.016), angina (p=0.023) and Framingham risk scores (p=0.011)	(18)F-NaF may be used for assessment of coronary artery plaque biology
32	Joshi et al., 2013 [43]	United Kingdom	80 patients with either MI or stable angina	Of the patients with MI (n=40), 93% had the highest coronary (18)F-NaF uptake in the culprit plaque compared to non-culprit (p<0.0001). There was no difference between culprit and non-culprit coronary (18)F-FDG uptake (p=0.34). Significant (18)F-NaF uptake occurred at all carotid plaque ruptures indicative of calcification, macrophage infiltration, apoptosis and necrosis	((18)F-NaF PET/CT is a non-invasive imaging technique to identify and localize both ruptured and high-risk plaques
33	Irkle et al., 2015 [44]	United Kingdom	7 patient’s Intimal atherosclerotic layers of human carotid arteries obtained from surgery	(18)F-NaF co-localized to calcified deposits on the plaque with both high affinity and selectivity. Areas of macro-calcification and micro-calcification were able to be deciphered by (18)F-NaF PET/CT imaging	Use of (18)F-NaF may allow for non-invasive detection of micro-calcification in unstable atherosclerosis
34	Vesey et al., 2017 [45]	United Kingdom	26 patients after TIA or stroke	(18)F-fluoride selectively localized to micro-calcifications, and carotid uptake was increased in culprit plaques compared to both asymptomatic contralateral plaques (p=0.001) and control patients (p=0.001). Uptake of (18)F-fluoride was significantly associated with high-risk plaque features and was a predictor of cardiovascular risk (p=0.003).	(18)F-fluoride PET/CT may be used to identify high-risk carotid plaques in patients
35	Chimowitz et al., 2011 [47]	USA	451 patients after TIA or stroke	30-day recurrent stroke rate or death was 14.7% in the percutaneous transluminal angioplasty and stenting (PTAS) group compared to 5.8% in the medical-management group (p=0.002). The probability of stroke in the artery of interest after 1-year was 20% in the PTAS cohort and 12.2% in the medical-management group (p=0.009)	Medical management had better outcomes in patients with intracranial arterial stenosis compared to PTAS
36	Chambers et al., 2005 [52]	Meta-analysis	5223 patients with asymptomatic carotid stenosis	Patients who underwent CEA had better overall results if they suffered from primary outcome- perioperative stroke, subsequent stroke, or death- (RR:0.69) or ipsilateral stroke (RR: 0.71) compared to those treated medically	CEA slightly reduced the risk for any stroke including ipsilateral stroke over three years
37	Muller et al., 2020 [54]	Meta-analysis	9753 patients with carotid stenosis	Transfemoral carotid artery stenting (TF-CAS) was associated with significantly increased risk of death or stroke in patients with symptomatic carotid stenosis than CEA. TF-CAS had significantly lower risk of MI, cranial nerve palsy, and hematoma compared to CEA. Asymptomatic patients had no significant increase in death or stroke between groups, but recurrent stenosis of 50-99% was significantly more prevalent after TF-CAS	TF-CAS presents an extra risk in patients with symptomatic carotid stenosis

## Conclusions

Carotid artery calcification is important in clinical practice because stenosis leads to high morbidity and mortality. The pathophysiology of CAC is an immediate inflammatory response that results in microcalcifications. Microcalcifications cause plaque instability, while macrocalcification causes plaque stabilization. Medial calcification is linked to calcium-phosphate imbalance and diabetes and chronic renal disease. These two calcifications have distinct risk factors, indicating a distinct etiology. Some studies show that increasing plaque calcification results in stenosis with minimal or no symptoms. Carotid artery calcification as a cerebrovascular risk indicator is still debated. Several methods exist to identify and characterize carotid plaque severity and shape. The initial ultrasound imaging study is quick and inexpensive. PET-CT imaging with (18)F-Sodium Fluoride (18F-NaF) is a newer technique that can detect new microcalcifications. Anticoagulants, statins, and lowering risk factors are used to treat carotid stenosis. Following medical management alone was found to be inferior in several studies. Patients over 70 were twice as likely to die or have a stroke 30 days after carotid stenting. Long-term, stenting is riskier than endarterectomy. However, both stenting and endarterectomy are effective in preventing strokes.
